# Case of Cyanosis

**Published:** 1854-08

**Authors:** Thomas W. Shaw

**Affiliations:** Pittsburgh, Pennsylvania


*Case of Cyanosis.* By Thomas W. Shaw, M. D., of Pittsburgh, Pennsylvania.

In this young man, Alexander S——, aged 23 years, the attention of his parents was first directed to the blue color of his skin when he was about two months old. At the age of one year he suffered from an affection of the brain, which resulted in partial hemiplegia of the right side. This paralysis continued through life. In early life he passed successfully through mild attacks of measles, scarlet fever and hooping-cough. With these exceptions he had been afflicted with no diseases, but had always been healthy. He was rather above the ordinary height, but of a delicate and slender form. The deep blue color was well marked on the whole surface of the body, and more particularly in the mucous membranes; the skin was always unnaturally cold; the extremities of the fingers and toes were bulbous and their nails much incurvated, and the conjunctivæ highly injected. Notwithstanding his lameness he was always able to walk about. The extremities on the paralytic side were almost as perfectly developed as those on the sound side, showing that nutrition had progressed nearly equally in them. Exercise of any kind, or emotions of the mind that would hurry the circulation, increased the intensity of the color of the skin, and rendered respiration difficult. His pulse was feeble, though otherwise healthy, and on putting the ear over the cardiac region a bruit de soufflet was distinctly audible. His appetite and digestion were invariably good, and his disposition cheerful.

During the last two years he had been more feeble than usual, and occasionally complained of vertigo and œdema of the feet and ankles. For several weeks previous to his death there was a tendency to hemorrhage from the gums, which were swollen and spongy, and slight ecchymosed spots appeared on the extremities.

About the 1st June, while in a crowded church, on a very warm day, he complained of being oppressed, and walked to the church door, and discharged from his stomach as near as could be ascertained about three pints of blood, which prostrated him very much, and led his physician to suppose him dying. In a short time he recovered sufficiently to be taken home, remaining in a very feeble condition for two weeks, occasionally discharging blood from the bowels, when he died.

The post mortem examination was made eight hours after death, in the presence and with the assistance of Dr. Courtney of Sharpsburgh, the physician of the deceased.

The heart was found *in situ*, and about the usual size, with the right ventricle hypertrophied, and having the appearance of a natural left ventricle. The walls of the left ventricle were thinner than those of the right usually are, and enclosed a very small cavity. The right auricle was considerably dilated, and the left preternaturally small, with the foramen ovale closed; the semilunar and tricuspid valves were studded with osseous deposits. The aorta arose from both ventricles, but principally from the right, the septum being imperfect between the ventricles; the pulmonary artery was impervious, there being no arteries found going to the lungs, except the bronchial; the pulmonary veins were quite small, while the venæ cavæ were greatly enlarged, but normal in their location. The blood on entering the right auricle passed to the right ventricle, thence through the aorta to be distributed, unoxygenated, to the different parts of the body, to be returned to the heart through the venæ cavæ and pulmonary veins. The lungs were found perfectly healthy in their texture, but highly discolored; the stomach was distended with a large quantity of black coagulated blood, and had its mucous coat softened and covered with patches of sanguineous effusion. The other abdominal viscera were healthy, except the liver, which was unusually large. The brain was not examined.

The majority of persons having this malformation die early; very few ever attain the age of adolescence. This patient lived till he was over twenty-three years of age in comparatively good health. The case, is remarkable, therefore, as well for the age attained by the subject as for the extent of the cardiac malformation.

